# Hypertensive Disorders of Pregnancy in Relation to Coffee and Tea Consumption: The Japan Environment and Children’s Study

**DOI:** 10.3390/nu13020343

**Published:** 2021-01-24

**Authors:** Yoko Kawanishi, Aiko Kakigano, Takashi Kimura, Satoyo Ikehara, Takuyo Sato, Takuji Tomimatsu, Tadashi Kimura, Hiroyasu Iso, on behalf of the Japan Environment and Children’s Study Group

**Affiliations:** 1 Department of Obstetrics and Gynecology, Osaka University Graduate School of Medicine, 2-2, Yamadaoka, Suita-shi, Osaka 565-0871, Japan; kawanishi@gyne.med.osaka-u.ac.jp (Y.K.); tomimatsu@gyne.med.osaka-u.ac.jp (T.T.); tadashi@gyne.med.osaka-u.ac.jp (T.K.); 2 Department of Obstetrics and Gynecology, National Cerebral and Cardiovascular Center, 6-1, Kisibeshinmachi, Suita-shi, Osaka 564-8565, Japan; ahayapyon1020@yahoo.co.jp; 3 Department of Public Health, Hokkaido University Graduate School of Medicine, North 15, West 7, Kita-Ku, Sapporo-Shi, Hokkaido 060-8638, Japan; kimura@med.hokudai.ac.jp; 4 Public Health, Department of Social Medicine, Osaka University Graduate School of Medicine, 2-2, Yamadaoka, Suita-shi, Osaka 565-0871, Japan; s-ikehara@pbhel.med.osaka-u.ac.jp; 5 Division of Community Health and Research, Osaka Women’s and Children’s Hospital, 840, Murodocho, Izumi-shi, Osaka 594-1101, Japan; satotaku@wch.opho.jp

**Keywords:** caffeine, coffee, hypertensive disorders of pregnancy, tea

## Abstract

Background: The association between coffee/tea intake and hypertensive disorders of pregnancy (HDP) remains unclear. This study aimed to investigate the association of caffeine, coffee, and tea intake during pregnancy with the risk of HDP. Methods: We assessed this association in 85,533 singleton pregnant women with live births in the Japan Environment and Children’s Study, a prospective cohort in Japan that included women from early pregnancy onward. Caffeinated and decaffeinated coffee and tea (green, oolong, and black) consumption during pregnancy was assessed using a validated food frequency questionnaire conducted at mid-pregnancy, and caffeine intake was calculated based on coffee and tea consumption. Multivariable logistic regression was used to assess the association with the risk of HDP. Results: HDP developed in 2222 women (2.6%). Caffeine intake was weakly associated with increased risk of HDP; the multivariable odds ratio of HDP for the highest versus the lowest quartile was 1.26 (95% confidence interval: 1.11, 1.43). Coffee drinkers of two or more cups per day showed a decreased risk compared with non-drinkers (multivariable odds ratio 0.79; 0.62, 0.99) even after adjustment for total caffeine intake. Tea consumption was not associated with the risk of HDP. Conclusions: Our study suggests that higher caffeine intake may increase HDP risk, while coffee drinkers had a lower risk. Further high-quality studies are needed to replicate these findings, and to elucidate if other substances in coffee may be protective against HDP.

## 1. Introduction

Coffee and tea are two of the most popular caffeine-containing beverages worldwide. Since caffeine crosses the placental barrier, the influence of coffee and tea consumption on pregnancy has been investigated, focusing on the association between caffeine intake and miscarriage, fetal growth restriction, small-for-gestational-age newborns, or preterm birth. Several prospective studies have shown that caffeine intake of >200–300 mg was associated with the risk of low birth weight or abortion [1,2,3,4]. The World Health Organization and Canada recommend a maternal caffeine intake of <300 mg/day [5], while the United States of America and some European countries recommend an intake of <200 mg/day [6]. However, coffee and tea contain numerous substances other than caffeine, and it is still unclear whether those substances in coffee or tea per se affect pregnancy.

Till now, a few studies have investigated the association between caffeine intake and the risk of hypertensive disorders of pregnancy (HDP), one of the most common medical conditions in the peripartum period and one of the leading causes of maternal/perinatal morbidity and mortality worldwide [7,8]. In these studies, caffeine intake was not associated with the risk of HDP [9,10]. Other studies showed that coffee intake was associated with null or decreased risk of HDP whereas tea consumption was associated with a higher risk of HDP [10,11]. The above studies were performed in Western countries, and they made no comment or analysis on types of tea. In Japan, pregnant women generally have less coffee and black tea but consume higher levels of green and oolong tea; the average number of cups per day were 0.14 cups of coffee, 0.14 cups of black tea, and 3.4 cups of green/oolong tea [12].

Coffee and tea contain substances other than caffeine and that might explain the associations of coffee and tea intake and the risk of HDP. Candidates for the beneficial substances in coffee are chlorogenic acids and polyphenols [13]. Chlorogenic acids, e.g., metabolites such as ferulic, caffeic, and quinic acids have antioxidant and antihypertensive effects [14]. Green, oolong, and black tea were all derived from the leaves of the same plant, Camellia sinensis. They are categorized according to oxidized levels (low for green tea, intermediate for oolong tea, and high for black tea) with varied contents and they contain little chlorogenic acids but other substances like flavonoid (high for green tea, intermediate for oolong tea, and low for black tea) [15,16]. Therefore, we attempted to examine the association between type-specific tea intake and the risk of HDP. We also examined the associations of decaffeinated coffee and black tea intake with the risk of HDP to explore the potential effects of substances other than caffeine.

The laboratory findings suggested that caffeine impaired placental angiogenesis, which could lead to HDP [17]. HDP is developed by abnormal placentation due to several genetic or immunological factors, resulting in reduced placental perfusion and hypoxia, thereby causing oxidative stress, inflammation, and apoptosis of the placental syncytium. These in turn lead to impaired placental angiogenesis through an imbalance between placental pro-angiogenic factors or vascular endothelial growth factor (VEGF)/placental growth factor (PlGF) and anti-angiogenic factors such as VEGF receptor 1 (VEGFR-1). The increased expression of soluble VEGFR-1 (sVEGFR-1) is associated with decreased PlGF and VEGF expression. Subsequently, sVEGFR-1 is released into the systemic circulation and causes systemic endothelial dysfunction [18,19]. It was reported that caffeine exposure to chicken embryos decreased VEGF and PlGF expression [17]. Green tea extract was reported to reduce the expression of VEGFR-1 in human umbilical cord vein epithelial cells [20]. There was, however, no report about the influence of extracts from coffee, oolong tea, or black tea on the placenta or umbilical cord epithelial cells. From the findings from previous epidemiological and laboratory studies, we hypothesized that caffeine is associated with the increased risk of HDP, and substances other than caffeine in coffee have protective effects but those in tea have adverse effects on HDP.

## 2. Materials and Methods

### 2.1. Study Design

The Japan Environment and Children’s Study (JECS) is a nationwide birth cohort study initiated in January 2011 that sought to elucidate the environmental factors that affect children’s health and development. In total, 103,099 pregnancies, 49,679 male partners, and 100,148 newborns were registered between January 2011 and March 2014. Details about the JECS can be found in a previous study [21,22]. Information on pregnancy, delivery, prenatal care, and lifestyle factors was obtained using a self-administered questionnaire and survey sheets for medical records. The self-administered questionnaire was collected at registration, during pregnancy, and 1 month after delivery. The survey sheets for medical records were sent to cooperating medical institutions, and research coordinators or medical staff transcribed the medical records at every trimester, during the perinatal period, and 1 month after delivery. The major nutritional profile from the diet was based on the self-administered questionnaire which was previously used and validated in the Japan Public Health Center-based Prospective Study for the Next Generation and was calculated by the JECS Program Office according to its original protocol [23]. The JECS was conducted following the Helsinki Declaration and other nationally valid regulations. All participants provided written informed consent. The JECS protocol was reviewed and approved by the Ministry of the Environment’s Institutional Review Board on Epidemiological Studies and by the Ethics Committees of all participating institutions (No. 100406001). The present study is based on the jecs-ag-20160424 dataset, which was released in June 2016 and revised in October 2016.

### 2.2. Study Population

**Figure 1:**
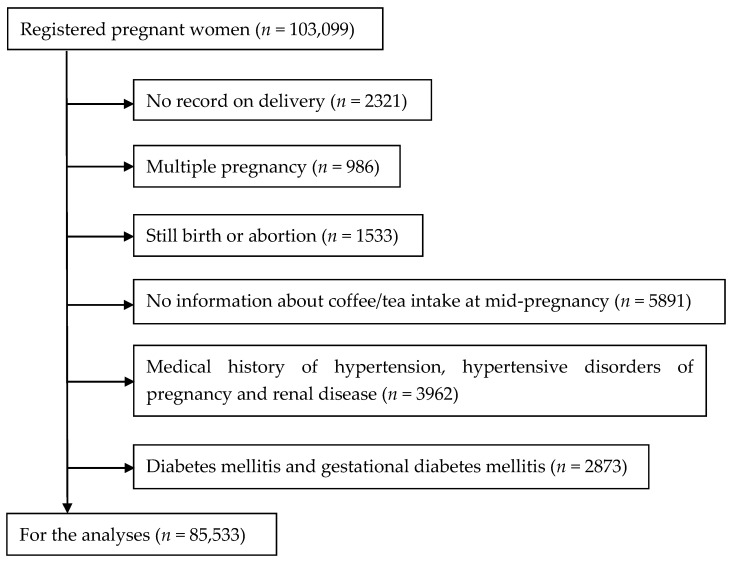
Flow chart for the study population.

We excluded cases without subsequent delivery records (*n* = 2321), cases of multiple pregnancies (*n* = 986), cases of stillbirth or abortion (*n* = 1533), and those who had incomplete information on coffee and tea intake during pregnancy (*n* = 5891). We also excluded cases with a medical history of hypertension, renal disease, history of HDP in previous pregnancies (*n* = 3962), and cases of diabetes mellitus and gestational diabetes mellitus (*n* = 2873). Consequently, 85,533 singleton pregnant women were included in the analyses (Figure 1).

### 2.3. Outcome Definition (HDP Classification)

During the registration period (2011–2014), pregnancy-induced hypertension at the time of the survey was defined according to the clinical guidelines of the Japan Society of Obstetrics and Gynecology and Japan Society for the Study of Hypertension in Pregnancy as newly diagnosed hypertension (diastolic blood pressure ≥ 90 mm Hg, systolic blood pressure ≥ 140 mm Hg, or both) with or without proteinuria (>300 mg in 24 h) from 20 weeks of gestation to 12 weeks after delivery [24,25]. The presence of both hypertension and proteinuria was defined as preeclampsia. We obtained information on HDP from the survey sheets from medical institutions at delivery and 1 month after delivery. Since we had no detailed information on proteinuria or hypertension, we could not distinguish HDP into gestational hypertension or preeclampsia.

### 2.4. Key Group Definitions (Assessment and Classification of Exposures)

Average daily consumption of coffee and tea was assessed using a questionnaire during the second or third trimester about their average frequencies after recognizing conception. Coffee intake included 3 serving styles: brewed, instant, and canned or bottled coffee. Green, oolong, and black tea intake included 2 serving styles: brewed and canned or bottled tea. The questionnaire regarding the consumption of each style of coffee, green tea, oolong tea, and black tea included multiple-choice questions with the following possible responses: “less than once a week,” “1 to 2 times per week,” “3 to 4 times per week,” “5 to 6 times per week,” “1 cup per day,” “2 to 3 cups per day,” “4 to 6 cups per day,” “7 to 9 cups per day,” and “10 cups or more per day”. To interpret the category of the servings per week or day, we allocated the median of each category 0, 1.5/7, 3.5/7, 5.5/7, 1, 2.5, 5, 8, and 10 servings per day, respectively.

Coffee and green, oolong, and black tea intakes were calculated as the sum of the intakes of each serving. Tea intake was defined as the sum of green, oolong, and black tea intakes. Caffeine intake was calculated as the sum of the caffeine contents in coffee, green tea, oolong tea, and black tea. The portion size of each beverage was determined as 120, 130, 120, and 150 mL for coffee, green tea, oolong tea, and black tea, respectively, using validated data from a previous Japanese prospective cohort study [12,26]. According to the Standard Tables of Food Composition in Japan 2015, 7th edition, caffeine content per 100 mL of each beverage is as follows: coffee, 60 mg; green tea, 20 mg; oolong tea, 20 mg; and black tea, 30 mg [27]. Subsequently, we weighted total caffeine intake, taking the frequency of decaffeinated coffee and black tea consumption into account. For those who reported “always” and “sometimes” consuming decaffeinated beverages, the caffeine intake from the beverages was weighted as 0 and 0.5, respectively. When they reported “never”, “never cared”, or “unknown” regarding decaffeinated beverages, their caffeine intakes from the beverages were weighted as 1.

### 2.5. Assessment of Covariates

We identified potential confounding variables by reviewing published data [28,29]. Data on pre-pregnancy height and weight were obtained from the survey sheet at registration, and the body mass index (BMI; weight (kg)/height (m)^2^) was calculated. Information on study unit, educational attainment, smoking status, and folic acid supplementation during pregnancy were obtained from the self-administered questionnaire during the second or third trimester. Data on age, birth weight, parity, and gestational age at delivery were obtained from the survey sheet during the perinatal period. We made categories as follows; parity (primiparous or multiparous); pre-pregnancy BMI (quintiles); smoking habits (current smoker or not); habitual alcohol intake during pregnancy (yes or no); folic acid supplementation during pregnancy (yes or no); and educational levels (university and higher degree or not). We used a dummy variable for missing data in the analysis because the proportions of the missing data were low (0.3–1.2%).

### 2.6. Statistical Analysis

Risk characteristics of participants according to quartiles of total caffeine intake were tested with a trend test using their median values. Coffee, tea, green tea, oolong tea, and black tea intake per day were categorized into four groups: none, <1 cup, 1 to <2 cups, and ≥2 cups. The lowest category for each variable was considered as the reference group. Multivariable logistic regression analysis was used to calculate the odds ratios (ORs) and 95% confidence intervals (CIs) of HDP according to each exposure; quantities of caffeine intake, categories of daily frequencies of coffee and tea intake, and frequencies of decaffeinated coffee and tea. We initially adjusted for age and study unit as basic model, wand we further adjusted for covariates as model 1.

For coffee and tea intake, we used two multivariable models. In model 1, to evaluate the effect of coffee and tea intakes separately, we adjusted for covariates and also used the categories of daily frequencies of coffee and tea intake for each other’s adjustments. In model 2, to evaluate the effects of substances in coffee and tea other than caffeine, we adjusted further for total caffeine intake.

We analyzed green, oolong, and black teas separately in the same way as coffee and tea, adjusting for covariates, coffee intake, and the three categories of tea intake with each other in model 1, and adjusting further for total caffeine intake in model 2.

For frequencies of decaffeinated coffee, as model 1, we adjusted for covariates, tea intake, and coffee intake weighted by the frequency of decaffeinated coffee. In model 2, we further adjusted for total caffeine intake. For frequencies of decaffeinated tea, as model 1, we adjusted for covariates, coffee, green tea, and oolong tea intakes, black tea intake weighted by the frequency of decaffeinated black tea. In model 2, we further adjusted for total caffeine intake.

To test for trends of ORs across the exposure categories, we modeled the median of each category of total caffeine, coffee, or tea intakes as a continuous variable. To confirm if there are trends of decaffeinated beverages, we used 0, 0.5, and 1 for the categories “never or unknown,” “sometimes”, and “always”, respectively. All statistical analyses were performed using SAS software version 9.4 (SAS Institute Inc., Cary, NC, USA). A *p*-value of <0.05 (two-tailed) was considered statistically significant.

## 3. Results

### 3.1. Characteristics of Participants According to Total Caffeine Intake

Out of the 85,533 singleton pregnant women, 2222 were diagnosed with HDP. The overall incidence of HDP was 2.6%. Table 1 shows the characteristics of the participants according to total caffeine intake. Compared with those in the lowest quartile, women in the higher quartile were more likely to be older, multiparous, less educated, have a higher mean pre-pregnancy BMI, and less likely to take folic acid supplements. Characteristics of the participants according to coffee, total tea, green tea, oolong tea, and black tea intakes are shown in Supplementary Tables S1 to S5. Most of these results were similar to those of total caffeine intake. The exceptions were inverse associations of age with total tea and oolong tea intakes, a positive association of primiparity with oolong tea intake, and a positive association of high education with black tea intake.

### 3.2. Risk of HDP According to Total Caffeine Intake

Table 2 shows the ORs of HDP according to the quartiles of total caffeine intake in basic model and model 1. The median caffeine intake was 0, 23, 56, and 131 mg per day across the lowest, second, third, and highest quartiles. In basic model, total caffeine intake was not associated with the risk of HDP. When primiparity was adjusted further for the basic model, total caffeine intake was positively associated with the risk of HDP in a dose–response fashion. The OR for the highest versus lowest quartiles of total caffeine intake was 1.35 (95% CI 1.19, 1.52), *p* for trend <0.001 (not shown in table). Total caffeine intake was positively associated with risk of HDP after adjustment further for other confounding variables. The OR for the highest versus lowest quartiles of caffeine intake in model 1 was 1.26 (95% CI: 1.11, 1.43), *p* for trend =0.004.

### 3.3. Risk of HDP According to Coffee and Tea Intakes

Table 3 shows the ORs of HDP according to the frequencies of coffee and tea intakes in basic model, model 1 and model 2. Nearly half of women did not consume coffee, and a quarter of women did not drink tea. Those who avoided both coffee and tea comprised 15% of the study participants. Higher frequency of coffee intake was associated with a reduced risk of HDP in basic model, which remained statistically significant even after adjusting for potential confounders, including total caffeine intake in model 2. The ORs according to the frequency of coffee intake in model 2 per day were as follows: none, 1.00; >0 and <1 cup, 0.92 (95% CI: 0.83, 1.01); 1 to <2 cups, 0.95 (95% CI: 0.83, 1.09); and ≥2 cups, 0.79 (95% CI: 0.62, 0.99); *p* for trend =0.012. A higher frequency of tea intake was associated with an increased risk in basic model and model 1, but this association of coffee and total tea intakes by cups per day was no longer statistically significant in model 2.

### 3.4. Risk of HDP According to Green, Oolong, and Black Tea Intakes

We conducted the subsequent analysis by mutually adjusting the frequencies of green, oolong, and black tea intakes (Table 4). The proportion of women who consumed green tea was the highest, followed by oolong and black tea. Higher frequencies of oolong and black tea intakes were associated with the increased risks of HDP in basic model. However, these associations were no longer statistically significant in model 2.

### 3.5. Risk of HDP According to the Frequencies of Decaffeinated Coffee and Tea Intakes

We also analyzed the associations between the frequencies of decaffeinated coffee and black tea intakes with the risk of HDP (Table 5). The frequency of decaffeinated coffee intake was associated with a decreased risk of HDP, and the trend was statistically significant in model 2. The frequency of decaffeinated black tea intake was not associated with the risk of HDP.

## 4. Discussion

In this large prospective study of approximately 85,500 pregnant women, a higher total caffeine intake during pregnancy was associated with a higher risk of HDP, after adjustment for potential confounding variables. After further adjustment for total caffeine intake, a higher frequency of coffee intake was associated with a reduced risk of HDP, whereas the frequencies of tea and each type of tea intakes were not associated with the risk of HDP.

Only few studies have investigated the association between caffeine intake and the risk of HDP. A prospective population-based cohort study of 7890 pregnant women showed no overall association between caffeine intake and the risk of HDP [9]. In that study, pregnant women who consumed 180–360 mg of caffeine per day tended to have a lower risk of preeclampsia: (OR: 0.79, 95% CI: 0.61, 1.01), however, those with a higher caffeine intake of >360 mg per day did not (OR: 1.03, 95% CI: 0.69, 1.55) after adjusting for maternal age, BMI, height, ethnicity, educational level, parity, alcohol consumption, smoking habits, folic acid supplement use, total daily energy intake, and stress during pregnancy. A population-based cohort study of 936 healthy pregnant women with uncomplicated pregnancy revealed that caffeine intake was not associated with the risk of HDP after adjusting for smoking and maternal age (OR: 1.05, 95% CI: 0.87, 1.27 for every 100 mg/day increase in caffeine intake) [10]. These results were not in line with our study. In our study, most of the participants consumed caffeine far below the amounts recommended by Western countries (200 mg) [5] and the World Health Organization (300 mg) [6], but caffeine intake consuming ≥6 mg per day was weakly associated with an increased risk of HDP. We should also note that in the basic model adjusting for age and study unit, the risk of HDP was null but after multivariable analysis, an increased risk was observed. This appeared that the association was due to a significant confounding by primiparity. Primiparity was inversely and strongly correlated with total caffeine intake and also was associated with a higher risk of HDP.

As we noted previously, caffeine exposure to chicken embryos, morphologically similar to the mammalian placenta, downregulated VEGF and PlGF [17] and hence, caffeine could disturb placental angiogenesis and increase the risk of HDP.

Coffee and tea intakes have shown different associations with the risk of HDP. In our study, coffee intake was associated with a decreased risk of HDP after adjusting for total caffeine intake, and the frequency of decaffeinated coffee intake tended to be associated with a decreased risk of HDP. In contrast, after adjusting for total caffeine intake, tea intake, three types of tea intake, and the frequency of decaffeinated black tea were not associated with the risk of HDP. As described earlier, in a cohort study of 936 women, coffee intake tended to be inversely associated with the risk of HDP (adjusted OR: 0.94, 95% CI: 0.77, 1.06 for a one-cup increase in coffee consumed) while tea intake (not specific for tea types) was associated with a higher risk of HDP (adjusted OR: 1.13, 95% CI: 1.04, 1.23 for a one-cup unit increase in tea consumed) [10]. A retrospective case–control study of 338 women, with 92 preeclamptic women and 245 normotensive women, also suggested that tea intake (not specific for tea types) during pregnancy was associated with a higher risk of preeclampsia after adjusting for maternal age, prior history of abortion, BMI, education, and smoking habit (OR: 1.88, 95% CI: 1.01, 3.51), while coffee intake was not associated with the risk of HDP (OR: 0.92, 95% CI: 0.54, 1.56) [11]. These findings on coffee and tea intakes were consistent with results when not adjusting for total caffeine intake. The present study in the first to suggest the inverse association between the frequency of decaffeinated coffee intake and the risk of HDP.

Our results support the notion that caffeine per se could increase the risk of HDP, and the inconsistency of findings on coffee and tea intakes may be explained by differences of substances in caffeine-containing beverages. For example, it is known that chlorogenic acids are rich in coffee, comprising about 3% in dry weight of roasted coffee powder [30], whereas chlorogenic acids in tea were reported to be 0.02–0.03% in dried weight of black, oolong, and green teas [31]. Green tea is known to be rich in catechins like epigallocatechin gallate, epigallocatechin, epicatechin, and epicatechin gallate [31]. These catechins are oxidized into tea polyphenols like theaflavins and theasinensins rich in black tea [30].

Our study had some strengths and limitations. This prospective cohort study had a large sample size, which covered approximately 3% of childbirths in Japan during the study period. To the best of our knowledge, this was the largest study on the association between caffeine, coffee, and tea intakes and the risk of HDP. Moreover, the diagnosis of HDP was based on survey sheets from medical institutions. The physicians were blinded to the information on exposure variables and therefore the diagnosis bias would have been small.

Regarding limitations, our study lacked the detailed information on HDP categories, such as gestational hypertension and preeclampsia. Second, no information was available on other beverages and foods, such as hot chocolate, soft drinks, energy drinks, or green tea-flavored sweets from the questionnaire. Third, we had no information on the frequency of decaffeinated green tea and oolong tea intakes, which had become more commonly merchandised in Japan after the administration of the questionnaire. Last, there could be residual confounding on unexamined confounding variables to examine the associations.

Conventionally, studies on coffee and tea intakes during pregnancy have focused on the safety threshold of caffeine intake. Although our study suggests that total caffeine intake during pregnancy may increase the risk of HDP in a dose–response fashion, coffee intake was associated with a decreased risk of HDP. Further studies are needed to examine the consistency of our results and the underlying mechanisms.

## Figures and Tables

**Table 1:** Risk characteristics of participants according to total caffeine intake.

	Caffeine Intake				
	Q1 (Low)	Q2	Q3	Q4 (High)	*p* for Trend
Number of subjects	21,422	21,306	21,517	21,288	
Median caffeine intake, mg per day	0	23	56	131	<0.001
Maternal age, year (SD)	31.1 (4.9)	30.7 (5.0)	31.1 (5.0)	31.3 (5.1)	<0.001
Gestational age at birth, week (SD)	39.3 (1.5)	39.3 (1.5)	39.3 (1.5)	39.3 (1.5)	0.001
Birth weight, kg (SD)	3027 (406)	3035 (409)	3036 (402)	3022 (411)	<0.001
Pre-pregnancy BMI, kg/m^2^ (SD)	20.9 (3.0)	21.1 (3.1)	21.1 (3.1)	21.2 (3.3)	<0.001
Primiparity, %	52.9	46.8	39.0	31.4	<0.001
Number of women with missing data	75	60	64	48	
High educational attainment, %	24.8	22.7	21.6	19.5	<0.001
Number of women with missing data	70	73	72	70	
Current drinkers during pregnancy, %	1.2	2.1	3.3	4.6	<0.001
Number of women with missing data	219	239	252	302	
Current smokers during pregnancy, %	2.2	3.1	4.8	8.0	<0.001
Number of women with missing data	110	125	137	122	
Folic acid supplementation, %	57	52.6	46.5	40.6	<0.001
Number of women with missing data	134	147	167	184	
Coffee intake, cups per day (SD)	0.20 (0.61)	0.24 (0.48)	0.46 (0.48)	1.03 (1.20)	<0.001
Total tea intake, cups per day (SD)	0.12 (0.30)	0.63 (0.40)	1.24 (0.89)	3.46 (2.99)	<0.001
Green tea intake, cups per day (SD)	0.04 (0.08)	0.35 (0.33)	0.75 (0.81)	2.27 (2.48)	<0.001
Oolong tea intake, cups per day (SD)	0.02 (0.07)	0.11 (0.21)	0.25 (0.49)	0.80 (1.82)	<0.001
Black tea intake, cups per day (SD)	0.05 (0.28)	0.16 (0.25)	0.24 (0.34)	0.39 (0.75)	<0.001

Standard Deviation (SD).

**Table 2:** ORs (95% CIs) of HDP according to quintiles of total caffeine intake in basic model and model 1.

	Q1 (Low)	Q2	Q3	Q4 (High)	*p* for Trend
Number of subjects	21,422	21,306	21,517	21,288	
Number of HDP	541	556	559	566	
Median caffeine intake, mg	0	23	56	131	
Rage of caffeine intake, mg	0–11	11–36	36–80	81–3628	
OR (95% CI) of basic model ^a^	1	1.04 (0.92, 1.17)	1.02 (0.91, 1.15)	1.04 (0.93, 1.18)	0.858
OR (95% CI) of model 1 ^b^	1	1.11 (0.98, 1.25)	1.15 (1.02, 1.30)	1.26 (1.11, 1.43)	0.004

Confidence intervals (CIs); hypertensive disorders of pregnancy (HDP); odds ratios (ORs). ^a^ Adjusted for age and study unit. ^b^ Adjusted further for parity, pre-pregnancy body mass index, smoking, alcohol consumption, folic acid supplementation, and education level.

**Table 3:** ORs (95% CIs) of HDP according to the frequency of coffee and total tea intakes by cups per day in basic model, model 1 and model 2.

	None	<1 Cup	1 to <2 Cups	≥2 Cups	*p* for Trend
Coffee					
Total number	38,483	29,684	12,258	5108	
Number of HDP	1068	741	303	110	
Range of caffeine intake, mg	0–1660	0–1137	0–859	0–3628	
Median of caffeine intake, mg	15	42	85	185	
OR (95% CI) of basic model ^a^	1	0.86(0.78, 0.95)	0.83(0.73, 0.94)	0.70(0.57, 0.86)	<0.001
OR (95% CI) of model 1 ^b^	1	0.93(0.85, 1.03)	0.99(0.87, 1.14)	0.89(0.72, 1.09)	0.063
OR (95% CI) of model 2 ^c^	1	0.92(0.83, 1.01)	0.95(0.83, 1.09)	0.79(0.62, 0.99)	0.012
Total Tea					
Total number	19,370	31,379	15,937	18,847	
Number of HDP	485	758	446	533	
Range of caffeine intake, mg	0–1080	0–1170	0–792	0–3628	
Median of caffeine intake, mg	0	22	47	125	
OR (95% CI) of basic model ^a^	1	0.98(0.87, 1.10)	1.13(0.99, 1.28)	1.15(1.02, 1.30)	0.007
OR (95% CI) of model 1 ^b^	1	1.05(0.94, 1.19)	1.23(1.07, 1.40)	1.24(1.09, 1.41)	<0.001
OR (95% CI) of model 2 ^c^	1	1.04(0.92, 1.17)	1.19(1.03, 1.36)	1.11(0.95, 1.29)	0.065

Confidence intervals (CIs); hypertensive disorders of pregnancy (HDP); odds ratios (ORs).^a^ Adjusted for age and study unit. ^b^ Adjusted further for parity, pre-pregnancy body mass index, smoking, alcohol consumption, folic acid supplementation, and education level. Coffee and tea intakes were mutually adjusted. ^c^ Adjusted further for total caffeine intake.

**Table 4:** ORs (95% CIs) of HDP according to the frequencies of green, oolong, and black tea intakes by cup per day in basic model, model 1 and model 2.

	None	<1 Cup	1 to <2 Cups	≥2 Cups	*p* for Trend
Green tea					
Total number	31,700	31,950	9505	12,378	
Number of HDP	789	828	253	352	
Range of caffeine intake, mg	0–1161	6–1170	26–2245	52–3628	
Median of caffeine intake, mg	8	34	59	130	
OR (95% CI) of basic model ^a^	1	1.02 (0.93, 1.13)	1.01 (0.88, 1.17)	1.10 (0.97, 1.26)	0.135
OR (95% CI) of model 1 ^b^	1	1.03 (0.93, 1.15)	1.03 (0.88, 1.20)	1.14 (1.00, 1.30)	0.023
OR (95% CI) of model 2 ^c^	1	1.02 (0.92, 1.14)	1.00 (0.86, 1.17)	1.05 (0.90, 1.23)	0.261
Oolong tea					
Total number	58,177	20,915	2863	3578	
Number of HDP	1439	584	86	113	
Range of caffeine intake, mg	0–1799	5–1501	24–2245	48–3628	
Median of caffeine intake, mg	26	44	71	138	
OR (95% CI) of basic model ^a^	1	1.19 (1.08, 1.32)	1.33 (1.06, 1.66)	1.42 (1.17, 1.73)	<0.001
OR (95% CI) of model 1 ^b^	1	1.13 (1.02, 1.25)	1.26 (1.00, 1.58)	1.31 (1.07, 1.60)	<0.001
OR (95% CI) of model 2 ^c^	1	1.12 (1.01, 1.24)	1.23 (0.98, 1.55)	1.21 (0.97, 1.51)	0.012
Black tea					
Total number	49,520	31,549	3382	1082	
Number of HDP	1308	796	79	39	
Range of caffeine intake, mg	0–1170	0–2245	0–1799	0–3628	
Median of caffeine intake, mg	21	46	91	169	
OR (95% CI) of basic model ^a^	1	0.99 (0.91, 1.09)	0.94 (0.74, 1.18)	1.42 (1.02, 1.96)	0.426
OR (95% CI) of model 1 ^b^	1	1.03 (0.94, 1.14)	0.97 (0.77, 1.24)	1.45 (1.04, 2.02)	0.272
OR (95% CI) of model 2 ^c^	1	1.02 (0.93, 1.13)	0.95 (0.75, 1.20)	1.33 (0.94, 1.88)	0.461

Confidence intervals (CIs); hypertensive disorders of pregnancy (HDP); odds ratios (ORs). ^a^ Adjusted for age and study unit. ^b^ Adjusted further for parity, pre-pregnancy body mass index, smoking, alcohol consumption, folic acid supplementation, education level, and coffee intake. Green, oolong, and black tea intake were mutually adjusted. ^c^ Adjusted further for total caffeine intake.

**Table 5:** ORs (95% CIs) of HDP according to the frequencies of decaffeinated coffee and tea intakes in basic model, model 1 and model 2.

	Never or Unknown	Sometimes	Always	*p* for Trend
Frequency of decaffeinated coffee				
Total number	54,764	21,683	9086	
Number of HDP	1431	573	218	
Range of caffeine intake, mg	0–3628	0–1137	0–746	
Median of caffeine intake, mg	46	32	11	
OR (95% CI) of basic model ^a^	1	0.97 (0.88, 1.07)	0.85 (0.73, 0.98)	0.052
OR (95% CI) of model 1 ^b^	1	0.93 (0.84, 1.03)	0.87 (0.75, 1.01)	0.008
OR (95% CI) of model 2 ^c^	1	0.93 (0.84, 1.03)	0.87 (0.75, 1.01)	0.019
Frequency of decaffeinated black tea intake				
Total number	64,822	17,750	2961	
Number of HDP	1682	459	81	
Range of caffeine intake, mg	0–3628	0–1029	0–746	
Median of caffeine intake, mg	41	27	11	
OR (95% CI) of basic model ^a^	1	1.01 (0.91, 1.13)	1.07 (0.85, 1.34)	0.763
OR (95% CI) of model 1 ^d^	1	0.98 (0.88, 1.10)	1.05 (0.83, 1.32)	0.753
OR (95% CI) of model 2 ^e^	1	0.98 (0.88, 1.09)	1.04 (0.83, 1.31)	0.738

Confidence intervals (CIs); hypertensive disorders of pregnancy (HDP); odds ratios (ORs). ^a^ Adjusted for age and study unit. ^b^ Adjusted further for parity, pre-pregnancy body mass index, smoking, alcohol consumption, folic acid supplementation, and education level, coffee intake weighted by the frequency of decaffeinated coffee intake, and tea intake. ^c^ Adjusted further for total caffeine intake. ^d^ Adjusted further for parity, pre-pregnancy body mass index, smoking, alcohol consumption, folic acid supplementation, and education level, coffee, green tea, and oolong tea intakes, black tea intake weighted by the frequency of decaffeinated black tea. ^e^ Adjusted further for total caffeine intake.

## Data Availability

Data sharing not applicable.

## Supplementary Materials

The following are available online at https://www.mdpi.com/2072-6643/13/2/343/s1, Table S1: Risk characteristics of participants according to the frequency of coffee intake, Table S2: Risk Characteristics of participants according to the frequency of tea intake, Table S3: Risk characteristics of participants according to the frequency of green tea intake, Table S4: Risk characteristics of participants according to the frequency of oolong tea intake, Table S5: Risk characteristics of participants according to the frequency of black tea intake.
