# Supplementary material for: Hypertensive Disorders of Pregnancy in Relation to Coffee and Tea Consumption: The Japan Environment and Children’s Study
Source: Nutrients. 2021 Jan 24;13(2):343. doi: 10.3390/nu13020343 (PMC7912571; doi:10.3390/nu13020343)
Supplement: Supplementary file 1 [file nutrients-13-00343-s001.pdf]

Table S1. Risk characteristics of participants according to the frequency of coffee intake.

	Coffee intake (per day)				
	None	<1 cup	1 to <2 cups	=>2 cups	*P* for trend
Number of subjects	38,483	29,684	12,258	5,108	
Maternal age, year (SD)	30.2 (5.1)	31.3 (4.9)	32.3 (4.6)	32.9 (4.5)	<0.001
Gestational age at birth, week (SD)	39.3 (1.5)	39.3 (1.5)	39.2 (1.5)	39.2 (1.5)	<0.001
Birth weight, kg (SD)	3,029 (408)	3,037 (405)	3,023 (411)	3,010 (407)	<0.001
Pre-pregnancy BMI, kg/m^2 (SD)	21.0 (3.1)	21.1 (3.2)	21.1 (3.0)	21.2 (3.1)	<0.001
Primiparity, %	51.7	40	29	20.6	<0.001
High educational attainment, %	22.6	22.6	22.1	17.1	<0.001
Current drinkers during pregnancy, %	1.5	3.1	4.5	6.4	<0.001
Current smokers during pregnancy, %	2.6	4.5	6.8	13.2	<0.001
Folic acid supplementation, %	50.4	49.9	46.4	42.5	<0.001
Total tea intake, cups per day (SD)	1.35 (2.15)	1.28 (1.79)	1.46 (1.93)	1.70 (2.50)	<0.001
Green tea intake, cups per day (SD)	0.85 (1.66)	0.79 (1.42)	0.93 (1.51)	1.07 (1.76)	<0.001
Oolong tea intake, cups per day (SD)	0.31 (1.08)	0.26 (0.86)	0.30 (0.94)	0.37 (1.19)	<0.001
Black tea intake, cups per day (SD)	0.19 (0.48)	0.22 (0.41)	0.23 (0.45)	0.26 (0.68)	<0.001

Standard Deviation (SD).

Table S2. Risk characteristics of participants according to the frequency of tea intake.

	Tea intake (per day)				
	None	<1 cup	1 to <2 cups	=>2 cups	*P* for
Number of subjects	19,370	31,379	15,937	18,847	
Maternal age, year (SD)	31.5 (4.8)	31.0 (4.9)	30.9(5.1)	30.9 (5.2)	<0.001
Gestational age at birth, week (SD)	39.3 (1.5)	39.3 (1.5)	39.3 (1.5)	39.3 (1.5)	0.306
Birth weight, kg (SD)	3,028 (412)	3,035 (403)	3,030 (411)	3,024 (406)	0.059
Pre-pregnancy BMI, kg/m^2 (SD)	20.9 (3.0)	21.1 (3.1)	21.1 (3.1)	21.2 (3.3)	<0.001
Primiparity, %	47.9	42.1	40.9	39.0	<0.001
High educational attainment, %	23.3	22.7	21.9	20.3	<0.001
Current drinkers during pregnancy, %	1.9	2.9	3.3	3.2	<0.001
Current smokers during pregnancy, %	3.2	4.t	4.9	5.5	<0.001
Folic acid supplementation, %	54.8	50.6	46.9	42.9	<0.001
Coffee intake, cups per day (SD)	0.39 (0.76)	0.47 (0.74)	0.52 (0.74)	0.57 (1.04)	<0.001
Green tea intake, cups per day (SD)	0 (0)	0.24 (0.22)	0.72 (0.39)	2.85 (2.35)	<0.001
Oolong tea intake, cups per day (SD)	0 (0)	0.09 (0.15)	0.26 (0.33)	0.98 (1.94)	<0.001
Black tea intake, cups per day (SD)	0 (0)	0.15 (0.18)	0.31 (0.33)	0.44 (0.86)	<0.001

Standard Deviation (SD).

Table S3. Risk characteristics of participants according to the frequency of green tea intake.

	Green tea intake (per day)				
	None	<1 cup	1 to <2 cups	=>2 cups	*P* for trend
Number of subjects	31,700	31,950	9,505	12,378	
Maternal age, year (SD)	31.0 (5.0)	30.9 (5.0)	31.3 (5.0)	31.4 (5.1)	<0.001
Gestational age at birth, week (SD)	39.3(1.5)	39.3(1.5)	39.3(1.5)	39.3(1.5)	0.026
Birth weight, kg (SD)	3,028 (406)	3,035 (408)	3,032 (411)	3,022 (405)	0.011
Pre-pregnancy BMI, kg/m^2 (SD)	21.0 (3.0)	21.2 (3.1)	21.2 (3.4)	21.1 (3.1)	<0.001
Primiparity, %	46.6	41.2	40.5	37.2	<0.001
High educational attainment, %	22.8	22.0	21.5	21.3	0.034
Current drinkers during pregnancy, %	2.3	3.1	3.2	3.1	<0.001
Current smokers during pregnancy, %	4.0	5.0	4.5	4.5	<0.001
Folic acid supplementation, %	52.9	48.8	55.6	43.4	<0.001
Coffee intake, cups per day (SD)	0.42 (0.78)	0.49 (0.75)	0.57 (0.94)	0.56 (0.98)	<0.001
Total tea intake, cups per day (SD)	0.39 (1.15)	0.93 (0.97)	1.73 (1.01)	4.67 (2.80)	<0.001
Oolong tea intake, cups per day (SD)	0. 26 (1.07)	0.30 (0.85)	0.31 (0.77)	0.38 (1.26)	<0.001
Black tea intake, cups per day (SD)	0.13 (0.38)	0.23 (0.40)	0.30 (0.49)	0.29 (0.72)	<0.001

Standard Deviation (SD).

Table S4. Risk characteristics of participants according to the frequency of oolong tea intake.

	Oolong tea intake (per day)				
	None	<1 cup	1 to <2 cups	=>2 cups	*P* for trend
Number of subjects	58,177	20,915	2,863	3,578	
Maternal age, year (SD)	31.6 (4.8)	30.1 (5.1)	29.2 (5.2)	28.9 (5,2)	<0.001
Gestational age at birth, week (SD)	39.3 (1.5)	39.3 (1.5)	39.3 (1.5)	39.3 (1.5)	0.002
Birth weight, kg (SD)	3,030 (406)	3,032 (412)	3,027 (401)	3,029 (400)	0.841
Pre-pregnancy BMI, kg/m^2 (SD)	21.0 (3.0)	21.4 (3.3)	21.3 (4.0)	21.3 (3.4)	<0.001
Primiparity, %	41.7	44.0	45.1	44.7	<0.001
High educational attainment, %	24.1	19.1	13.9	15.2	<0.001
Current drinkers during pregnancy, %	2.6	3.1	2.9	3.2	<0.001
Current smokers during pregnancy, %	3.7	6.0	7.6	7.8	<0.001
Folic acid supplementation, %	51.1	46.9	40.0	38.8	<0.001
Coffee intake, cups per day (SD)	0.47 (0.79)	0.50 (0.77)	0.63 (1.22)	0.55 (1.17)	<0.001
Total tea intake, cups per day (SD)	1.02 (1.69)	1.40 (1.44)	2.42 (1.67)	5.79 (3.91)	<0.001
Green tea intake, cups per day (SD)	0.84 (1.61)	0.80 (1.33)	0.94 (1.35)	1.22 (2.15)	<0.001
Black tea intake, cups per day (SD)	0.18 (0.43)	0.26 (0.42)	0.38 (0.60)	0.35 (0.97)	<0.001

Standard Deviation (SD).

Table S5. Risk characteristics of participants according to the frequency of black tea intake

	Black tea intake (per day)				
	None	<1 cup	1 to <2 cups	=>2 cups	*P* for trend
Number of subjects	49,520	31,549	3,382	1,082	
Maternal age, year (SD)	31.1 (5.0)	31.0 (5.0)	31.2 (5.1)	31.7 (4.9)	0.003
Gestational age at birth, week (SD)	39.3 (1.5)	39.3 (1.5)	39.3 (1.5)	39.2 (1.8)	0.053
Birth weight, kg (SD)	3,029 (409)	3,034 (403)	3,027 (411)	3,011 (441)	0.143
Pre-pregnancy BMI, kg/m^2 (SD)	21.1 (3.1)	21.1 (3.2)	21.1 (3.2)	21.5 (3.5)	<0.001
Primiparity, %	44.0	40.4	37.4	32.0	<0.001
High educational attainment, %	20.9	23.5	25.3	25.4	<0.001
Current drinkers during pregnancy, %	2.5	3.2	3.3	4.5	<0.001
Current smokers during pregnancy, %	4.4	4.7	4.7	8.3	<0.001
Folic acid supplementation, %	49.3	49.2	47.2	45.2	0.008
Coffee intake, cups per day (SD)	0.45 (0.78)	0.52 (0.79)	0.59 (0.98)	0.78 (1.86)	<0.001
Total tea intake, cups per day (SD)	1.08 (1.93)	1.49 (1.72)	2.83 (2.17)	5.76 (4.64)	<0.001
Green tea intake, cups per day (SD)	0.81 (1.61)	0.85 (1.42)	1.24 (1.65)	1.82 (2.54)	<0.001
Oolong tea intake, cups per day (SD)	0.27 (1.01)	0.29 (0.88)	0.50 (1.21)	0.78 (1.99)	<0.001

Standard Deviation (SD).
